# EGF +61A/G polymorphism contributes to increased gastric cancer risk: evidence from a meta-analysis

**DOI:** 10.1186/s12935-014-0134-4

**Published:** 2014-12-12

**Authors:** Qiliu Peng, Shan Li, Xue Qin, Xianjun Lao, Zhiping Chen, Xiaolian Zhang, Junqiang Chen

**Affiliations:** Department of Clinical Laboratory, First Affiliated Hospital of Guangxi Medical University, Nanning, Guangxi China; Department of Occupational Health and Environmental Health, School of Public Health at Guangxi Medical University, Nanning, Guangxi China; Department of Gastrointestinal Surgery, First Affiliated Hospital of Guangxi Medical University, Nanning, 530021 Guangxi China

**Keywords:** Epidermal growth factor, Gastric cancer, Polymorphism, Meta-analysis

## Abstract

**Background:**

Epidermal growth factor (EGF) plays a pivotal role in cell proliferation, differentiation, and tumorigenesis of epithelial tissues. Variation of the EGF +61A/G (rs4444903) can lead to an alteration in EGF production and/or activity, which may result in individual susceptibility to gastric cancer. Studies investigating the association between EGF +61A/G polymorphism and gastric cancer risk produced inconsistent results. The aim of this study was to quantitatively summarize the evidence for such an association.

**Methods:**

Eligible studies on the association between EGF +61A/G polymorphism and gastric cancer risk were identified by search of electronic databases including PubMed, EMBASE, Cochrane Library, and Chinese Biomedical Literature database (CBM). Data were extracted by two independent authors and pooled odds ratios (ORs) with 95% confidence intervals (CIs) were used to assess the strength of the association. Metaregression and subgroup analyses were performed to identify the source of heterogeneity.

**Results:**

Finally, six case–control studies with 1547 gastric cancer cases and 2762 controls were eventually identified. Overall, significant increased gastric cancer risk was found when all studies were pooled in the meta-analysis (GG vs. AA: OR = 1.438, 95% CI 1.021–2.025, *P* = 0.038; GG + AG vs. AA: OR = 1.256, 95% CI 1.025–1.539, *P* = 0.028; GG vs. AG + AA: OR = 1.265, 95% CI 1.002–1.596, *P* = 0.048). In subgroup analysis by ethnicity, source of control, study quality, and HWE in controls, significant increased gastric cancer risk was observed in Asians, population-based studies, high quality studies, and studies consistent with HWE. In subgroup analysis according to tumor location, and histological type, significant association was observed in all subgroups.

**Conclusions:**

This meta-analysis suggested that the EGF +61A/G polymorphism contributes to increased gastric cancer risk, especially in Asian populations. Further well-designed studies based on large sample size in diverse populations are needed to confirm this association.

## Introduction

Gastric cancer is one of the most common cancers and the second leading cause of cancer-related death in the world [1,2]. Despite the decline in the number of cases in some western countries, the incidence of gastric cancer remains high in Eastern Asia [2]. Aetiologically, carcinogenesis of gastric cancer is a complex, multistep and multifactor process, in which many factors are involved. It has been well established that Helicobacter pylori (*H. pylori*) infection was the major risk factor for gastric cancer [3,4]. Additionally, some other factors including high consumption of salty food, low consumption of fresh fruits and vegetables, tobacco smoking, alcohol drinking are also considered as common risk factors [5,6]. However, most subjects with the above environmental risk factors never develop gastric cancer while many gastric cancer cases develop among individuals without the risk factors, suggesting that other factors such as genetic factors also play an important role in gastric carcinogenesis.

Epidermal growth factor (EGF) is a member of the EGF superfamily, which also includes transforming growth factor-α, heparin-binding EGF-like growth factor, epiregulin, betacellulin and amphiregulin [7]. As a growth factor, EGF has many biological functions, such as stimulation of DNA synthesis, proliferation, differentiation, and tumorigenesis of the epidermal tissues through binding with its receptor (EGFR) [8-10]. EGF is encoded by a 4.8 kb mRNA transcript from a 110 kb gene located on human chromosome 4q25–27. It was reported that a common single nucleotide change with a Guanine (G) to adenine (A) substitution at the position +61 in the 5′-untranslated region of the EGF gene (rs4444903) influences EGF production or protein expression by affecting DNA folding or gene transcription [11]. The +61GG and +61AG genotypes were correlated with significant higher expression of EGF than the +61AA genotype in peripheral blood mononuclear cell lines [11]. Moreover, the +61G allele had been found associated with increased promoter transcriptional activity than the +61A allele [12,13].

In light of the important biological function of the EGF +61A/G polymorphism, emerging epidemiological studies have been performed to investigate the association of EGF +61A/G polymorphism with gastric cancer risk, but the results remain inconsistent and underpowered. Some studies suggested that EGF +61A/G polymorphism was associated with an increased susceptibility to gastric cancer [14-17]. However, other studies failed to confirm such an association [18,19]. For genetic association studies that checked candidate polymorphisms, sample size is an important influencing factor for study accuracy [20]. Small sample size has insufficient power to detect a true association of modest effect, especially for the complex multifactorial disease such as gastric cancer. While combining data from all eligible studies by meta-analysis has the advantage of increasing statistical power and reducing random error and obtaining precise estimates for some potential genetic associations [21]. Therefore, in this study, we conducted a quantitative meta-analysis including all eligible studies. This is, to our knowledge, the first comprehensive meta-analysis of genetic studies on the association between EGF +61A/G polymorphism and gastric cancer.

## Materials and methods

### Literature search

A comprehensive literature search in Pubmed, Embase, Cochrane library, and CBM was conducted using the following combined keywords: ‘EGF’, ‘epidermal growth factor’, ‘polymorphism’, ‘SNP’, ‘genetics’, and ‘gastric cancer’. The latest search was performed in September 2014. There was no restriction on time period, sample size, population, language, or type of report. Additional studies were identified by a hand search of the references cited in the reviews and the retrieved articles. In addition, we also used the “Related Articles” function in PubMed to search other potential eligible studies. If more than one study was published using the same or overlapped data, only the study with the largest sample size was selected. The study was performed according to the proposal of Meta-analysis of Observational Studies in Epidemiology group (MOOSE) [22].

### Inclusion and exclusion criteria

Studies included in the meta-analysis were required to meet the following criteria: (1) case–control or cohort studies which investigated the association of EGF +61A/G polymorphism with gastric cancer; (2) provided an odds ratio (OR) with 95% confidence interval (CI) or other information for estimating OR (95% CI); and (3) the control group did not include malignant tumor patients. Studies were excluded if one of the following existed: (1) duplicate of previous publication; (2) no control population; (3) insufficient information for data extraction; and (4) case reports, conference abstracts, reviews, editorials, and letters.

### Quality assessment

Two authors independently assessed the quality of the studies by scoring according to the predetermined criteria (Table 1) which was modified from our previous study on gastric cancer [23]. The modified criteria included the representativeness of cases, ascertainment of gastric cancer, source of controls, quality control of genotyping methods, sample size, and Hardy-Weinberg equilibrium (HWE) in the control population. Studies with quality scores equal to or higher than 6 were considered as “high-quality” studies, whereas studies with scores less than 6 were considered as “low-quality” studies. Disputes were resolved through discussion.

**Table 1:** **Scale for quality assessment**

**Criteria**	**Score**
Representativeness of cases	
Selected from cancer registry or multiple cancer center sites	2
Selected from oncology department or cancer institute	1
Selected without clearly defined sampling frame or with extensive inclusion/exclusion criteria	0
Source of controls	
Population or community based	2
Both population-based and hospital-based/healthy volunteers/blood donors	1.5
Hospital-based controls without gastric cancer	1
Cancer-free controls without total description	0.5
Not described	0
Ascertainment of gastric cancer	
Histologically or pathologically confirmed	2
Diagnosis of gastric cancer by patient medical record	1
Not described	0
Sample size	
>1000	2
200-1000	1
<200	0
Quality control of genotyping methods	
Clearly described a different genotyping assay to confirm the data	1
Not described	0
Hardy-Weinberg equilibrium	
Hardy-Weinberg equilibrium in controls	1
Hardy-Weinberg disequilibrium in controls	0.5
No checking for Hardy-Weinberg disequilibrium	0

### Data extraction

Two authors (Xiaolian Zhang and Xianjun Lao) independently extracted data and reached consensus on all of the items. For each study, the following information was sought: first author, year of publication, country of origin, ethnicity of the study population, numbers of cases and controls, genotyping methods, matching criteria, source of control, ascertainment of cases, and distribution of genotypes and alleles in both groups. The tumor location and histological type of the gastric cancer cases were additionally recorded for the stratified analysis. When the genotype frequencies in a study were not provided, we contacted the authors to get the relevant information by e-mail or telephone.

### Statistical analysis

We assessed HWE in the controls for each study using a goodness-of-fit test (chi-square or Fisher’s exact test), and a P < 0.05 was considered as significant disequilibrium. The strength of the association between EGF +61A/G polymorphism and gastric cancer was estimated using ORs with the corresponding 95% CIs. The pooled ORs were performed for codominant model (GG vs. AA, AG vs. AA), dominant model (GG + AG vs. AA) and recessive model (GG vs. AG + AA).

The Chi-square-based Q test was used to assess the statistical heterogeneity among studies [24]. If the result of the *Q* test was *P*_*Q*_ < 0.10, suggesting the existence of heterogeneity, the pooled ORs were calculated using the random-effects model (the DerSimonian and Laird method) [25]. Otherwise, when the result of the *Q* test was *P*_*Q*_ ≥ 0.1, indicating the absence of heterogeneity, the fixed-effects model (the Mantel–Haenszel method) [26] was used. To identify the sources of heterogeneity across studies, we performed logistic meta-regression analysis and subgroup analyses. The following parameters were included as covariates in the meta-regression analysis: ethnicity (Asians vs. Caucasians), genotyping methods (PCR-RFLP vs. not PCR-RFLP), source of controls (HB vs. PB), HWE in the controls (Yes vs. No), and matched controls (yes vs. no). Subgroup analyses were performed according to ethnicity, source of control, study quality, HWE in controls, tumor location, and histological type.

Sensitivity analysis was performed by sequentially excluded the individual studies to assess the robustness of the results. Begg’s funnel plot and Egger’s regression asymmetry test were performed to evaluate the publication bias [27]. If the publication bias presented, the Duval and Tweedie non-parametric “trim and fill” method was applied to adjust for it [28]. All P values were two-sided. All analyses were performed using Stata software, version 12.0 (Stata Corp., College Station, TX).

## Results

### Characteristics of studies

**Figure 1:**
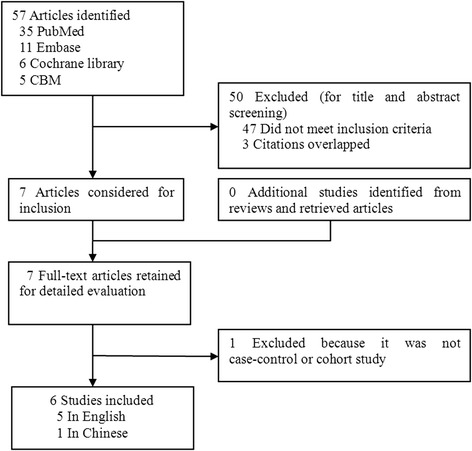
**Flowchart for study selection in the meta-analysis.**

Based on the search strategy, seven studies evaluating the EGF +61A/G polymorphism and gastric cancer susceptibility were identified. One of these articles was excluded because it was not case–control or cohort study [29]. Manual search of references cited in the reviews and the retrieved articles did not found any additional studies. As a result, six case–control studies with 1547 gastric cancer cases and 2762 controls were eventually included in the meta-analysis (Figure 1). Table 2 lists the main characteristics of these studies. Among these studies, five were conducted in Asian descent [14-18] and one was conducted in Caucasian descent [19]. Two were population–based studies [15,18] and four were hospital–based studies [14,16,17,19]. All studies used validated methods including PCR-RFLP, PCR-CTPP to genotype the EGF +61A/G polymorphism. The gastric cancer cases were histologically or pathologically confirmed in five of the eligible studies [15-19]. The genotype distribution of the control group in one study was inconsistent with HWE [19].

**Table 2:** **Characteristics of studies included in the meta-analysis**

**First author (Year)**	**Country**	**Ethnicity**	**Sample size (case/control)**	**Genotyping methods**	**Matching criteria**	**Source of control**	**GC ascertainment**	**Quality scores**	**HWE (** ***P*** **value)**
Hamai 2004	Japan	Asian	200/230	PCR-RFLP	Age, sex, and *H. pylori* infection	HB	NR	5.5	0.647
Goto 2005	Japan	Asian	202/450	PCR-CTPP	NR	PB	Pathologically confirmed	6.5	0.537
Jin 2007	China	Asian	617/660	PCR-RFLP	Age, sex, smoking and drinking	PB	Histopathologically confirmed	7.0	0.407
Araujo 2011	Portugal	Caucasian	207/984	PCR-RFLP	NR	HB	Histologically confirmed	4.5	0.010
Yang 2012	China	Asian	207/318	PCR-RFLP	Age, and sex	HB	Histologically confirmed	6.5	0.272
Lin 2012	China	Asian	114/120	PCR-RFLP	Sex	HB	Pathologically confirmed	5.0	0.485

GC, Gastric cancer; NR, Not reported; PB, Population–based; HB, Hospital–based; HWE, Hardy–Weinberg equilibrium in control population; PCR–RFLP, Polymerase chain reaction-restriction fragment length polymorphism; PCR-CTPP, PCR with confronting two-pair primers; *H. pylori,* Helicobacter pylori.

### Quantitative synthesis

**Figure 2:**
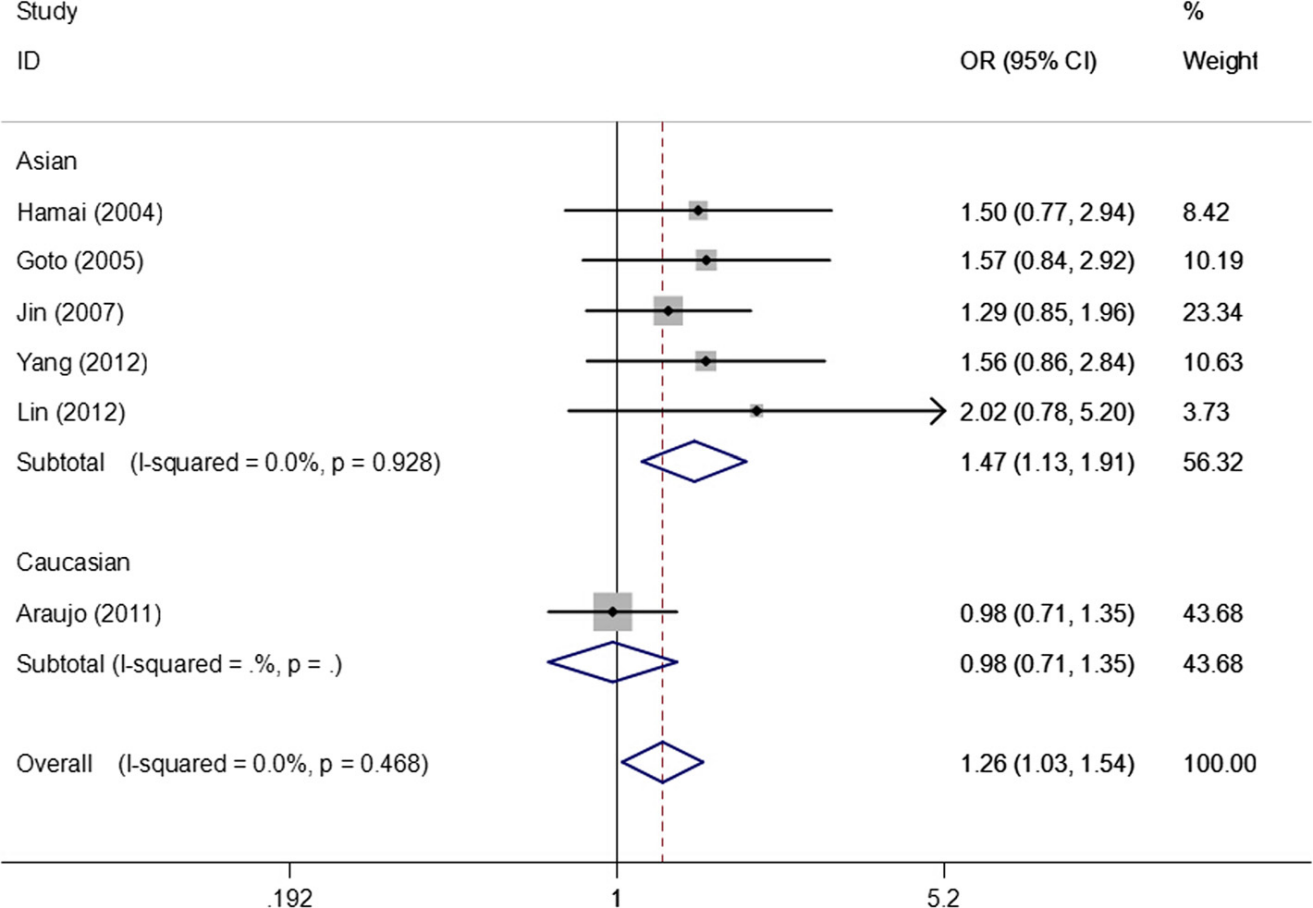
**Forest plots of EGF +61A/G polymorphism and gastric cancer risk in subgroup analysis by ethnicity using a fixed-effect model (dominant model GG + AG vs. AA).**

**Figure 3:**
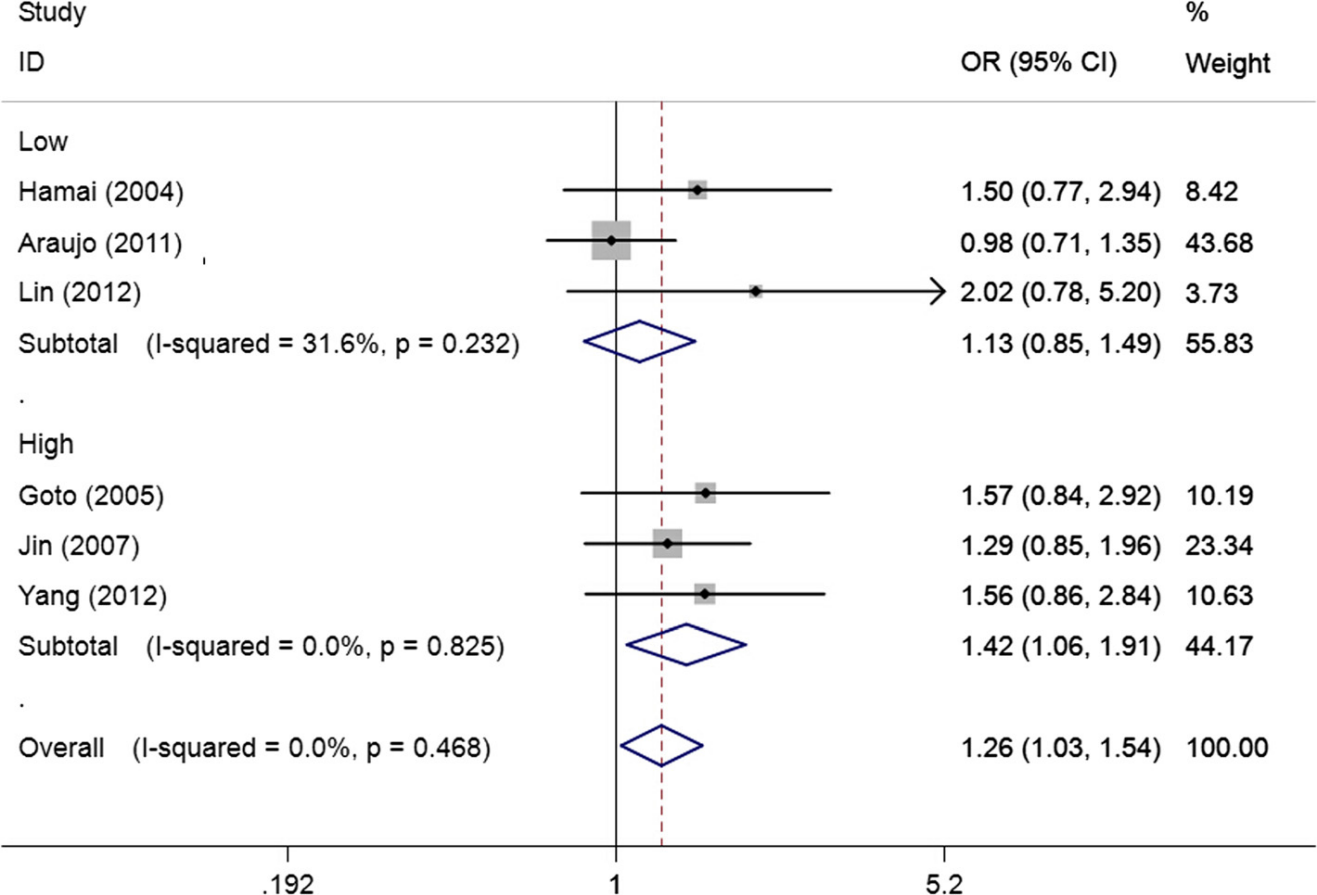
**Forest plots of EGF +61A/G polymorphism and gastric cancer risk in subgroup analysis by study quality using a fixed-effect model (dominant model GG + AG vs. AA).**

**Figure 4:**
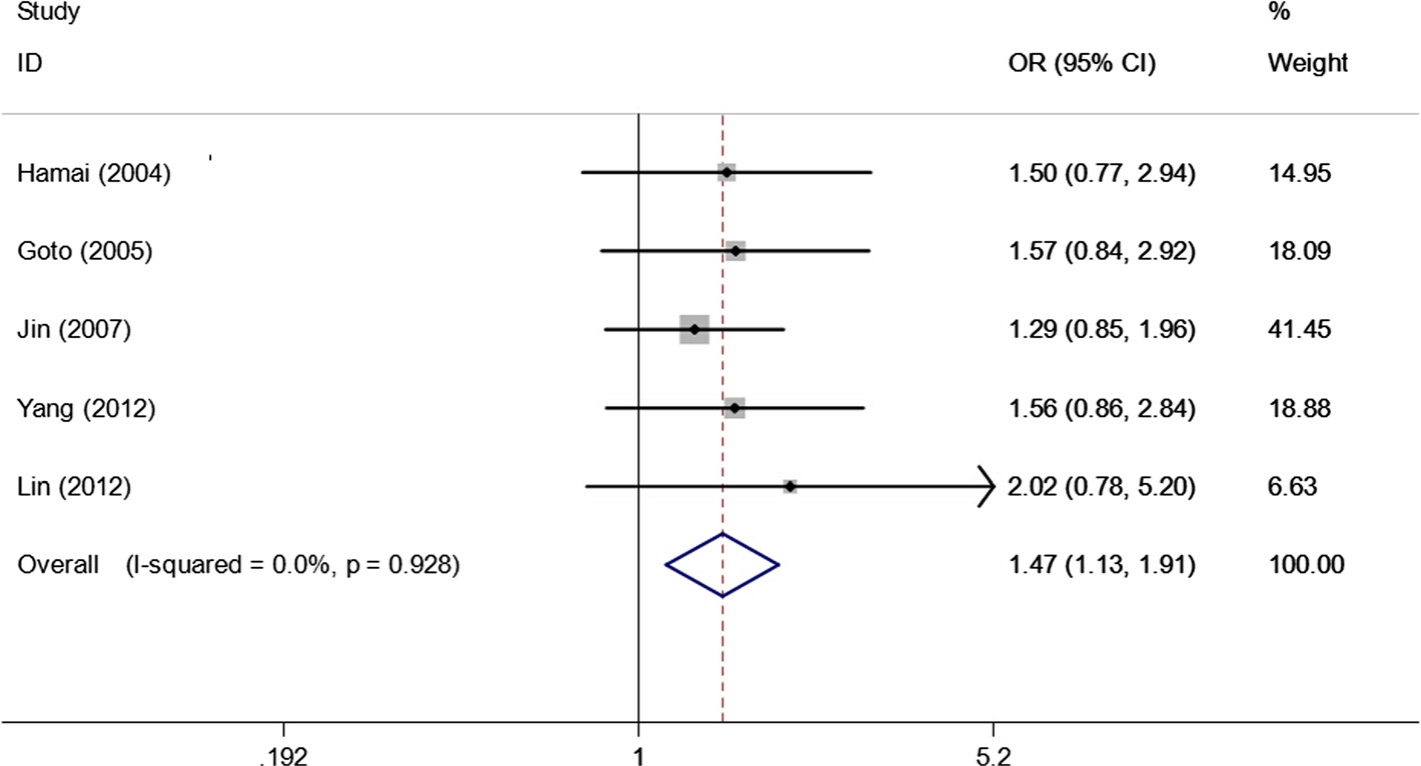
**Forest plots of EGF +61A/G polymorphism and gastric cancer risk in studies consistent with HWE using a fixed-effect model (dominant model GG + AG vs. AA).**

As shown in Table 3, we found that the EGF +61A/G polymorphism was significantly correlated with increased gastric cancer risk when all studies were pooled into the meta-analysis (GG vs. AA: OR = 1.438, 95% CI 1.021–2.025, *P* = 0.038; GG + AG vs. AA: OR = 1.256, 95% CI 1.025–1.539, *P* = 0.028; GG vs. AG + AA: OR = 1.265, 95% CI 1.002–1.596, *P* = 0.048). In subgroup analysis by ethnicity, significant increased gastric cancer risk was found in Asian populations (GG vs. AA: OR = 1.658, 95% CI 1.265–2.173, *P* = 0.000; GG + AG vs. AA: OR = 1.473, 95% CI 1.134–1.914, *P* = 0.004, Figure 2; GG vs. AG + AA: OR = 1.355, 95% CI 1.174–1.564, *P* = 0.000), but not in Caucasian populations. In stratified analysis according to source of control, significant increased gastric cancer risk was observed in population-based studies (GG vs. AA: OR = 1.477, 95% CI 1.035–2.108, *P* = 0.031; GG vs. AG + AA: OR = 1.220, 95% CI 1.016–1.466, *P* = 0.033), but not in hospital-based studies. In subgroup analysis by study quality, significant increased gastric cancer risk was observed in high quality studies (GG vs. AA: OR = 1.552, 95% CI 1.140–2.112, *P* = 0.005; GG + AG vs. AA: OR = 1.421, 95% CI 1.055–1.915, *P* = 0.021, Figure 3; GG vs. AG + AA: OR = 1.267, 95% CI 1.077–1.491, *P* = 0.004), but not in low quality studies. In stratified analysis by HWE in controls, significant increased gastric cancer risk was found in studies consistent with HWE (GG vs. AA: OR = 1.658, 95% CI 1.265–2.173, *P* = 0.000; GG + AG vs. AA: OR = 1.473, 95% CI 1.134–1.914, *P* = 0.004, Figure 4; GG vs. AG + AA: OR = 1.355, 95% CI 1.174–1.564, *P* = 0.000), but not in studies inconsistent with HWE. In subgroup analyses according to tumor location and histological type, significant association was observed in all subgroups.

**Table 3:** **Meta-analysis of EGF +61A/G polymorphism and gastric cancer risk**

**Analysis**	**No. of studies**	**GG vs. AA (Homozygote)**		**AG vs. AA (Heterozygote)**		**GG + AG vs. AA (Dominant model)**		**GG vs. AG + AA (Recessive model)**	
		**OR (95% CI)**	***P/P***_***Q***_	**OR (95% CI)**	***P/P***_***Q***_	**OR (95% CI)**	***P/P***_***Q***_	**OR (95% CI)**	***P/P***_***Q***_
Overall	6	1.438(1.021-2.025)	0.038/0.074	1.193(0.963-1.478)	0.106/0.927	1.256(1.025-1.539)	0.028/0.468	1.265(1.002-1.596)	0.048/0.020
Ethnicity									
Asian	5	1.658(1.265-2.173)	0.000/0.834	1.269(0.964-1.670)	0.090/0.924	1.473(1.134-1.914)	0.004/0.928	1.355(1.174-1.564)	0.000/0.318
Caucasian	1	0.769(0.498-1.189)	0.238/—	1.083(0.769-1.526)	0.647/—	0.977(0.707-1.349)	0.886/—	0.733(0.500-1.073)	0.110/—
Source of control									
HB	4	1.486(0.835-2.647)	0.178/0.023	1.156(0.885-1.510)	0.286/0.901	1.196(0.929-1.538)	0.164/0.272	1.328(0.882-2.000)	0.174/0.006
PB	2	1.477(1.035-2.108)	0.031/0.836	1.262(0.882-1.806)	0.204/0.415	1.377(0.976-1.942)	0.068/0.616	1.220(1.016-1.466)	0.033/0.357
Study quality									
High	3	1.552(1.140-2.112)	0.005/0.847	1.272(0.931-1.738)	0.131/0.714	1.421(1.055-1.915)	0.021/0.825	1.267(1.077-1.491)	0.004/0.450
Low	3	1.425(0.659-3.081)	0.368/0.021	1.127(0.839-1.512)	0.427/0.813	1.126(0.853-1.487)	0.403/0.232	1.294(0.714-2.344)	0.396/0.003
HWE									
Yes	5	1.658(1.265-2.173)	0.000/0.834	1.269(0.964-1.670)	0.090/0.924	1.473(1.134-1.914)	0.004/0.928	1.355(1.174-1.564)	0.000/0.318
No	1	0.769(0.498-1.189)	0.238/—	1.083(0.769-1.526)	0.647/—	0.977(0.707-1.349)	0.886/—	0.733(0.500-1.073)	0.110/—
Tumor location									
Cardia	3	1.322(1.041-1.987)	0.013/0.632	1.198(0.946-1.973)	0.238/0.322	1.356(1.013-1.966)	0.017/0.390	1.156(1.025-2.491)	0.022/0.374
Non-cardia	2	1.253(1.019-3.294)	0.028/0.388	1.159(0.847-1.869)	0.389/0.476	1.137(0.753-2.697)	0.528/0.387	1.297(1.009-2.984)	0.046/0.454
Histological type									
Intestinal	2	1.316(1.013-2.231)	0.016/0.671	1.065(0.789-1.842)	0.357/0.412	1.201(1.083-1.978)	0.013/0.721	1.092(0.992-2.257)	0.054/0.357
Diffuse	2	1.240(1.072-3.013)	0.022/0.336	1.103(0.817-1.627)	0.465/0.511	1.212(0.971-1.845)	0.066/0.348	1.115(1.008-2.327)	0.041/0.303

EGF, epidermal growth factor; *P*_*Q*_, P values of Q-test for heterogeneity test; OR, odds ratio; CI, confidence intervals; HB, Hospital–based studies; PB, Population-based studies; HWE, Hardy–Weinberg equilibrium in control population.

### Heterogeneity analysis

Statistical significant heterogeneity among studies was observed in the association analysis between the EGF +61A/G polymorphism and gastric cancer risk in the overall populations (GG vs. AA: *P*_*Q*_ = 0.074; GG vs. AG + AA: *P*_*Q*_ = 0.048; Table 3). To identify the sources of heterogeneity across studies, we first performed subgroup analyses. Subgroup analyses by source of controls and study quality revealed that the heterogeneity was still evident in hospital-based studies and low quality studies. Subsequently, we performed meta-regression analysis to further identify the source of heterogeneity. Meta-regression analysis indicated that the HWE in controls was the major source which contributed to the heterogeneity. When we excluded the HWE-violating study [19], the heterogeneity disappeared in both the overall populations and subgroup analyses. However, the significance of the pooled ORs in different comparison models the overall populations and subgroup analyses were not influenced by excluding this study [19] (Table 3).

### Sensitivity analysis

Sensitivity analysis was performed to assess the influence of each individual study on the pooled ORs by sequential removal of individual studies. The results revealed that no individual study significantly affected the pooled ORs. In addition, sensitivity analysis was further performed by excluding the HWE-violating study [19] and the study without definite ascertainment for gastric caner patients [14]. The significance of pooled ORs was not altered after excluding the two studies, indicating that our results were robust and reliable.

### Publication bias

**Figure 5:**
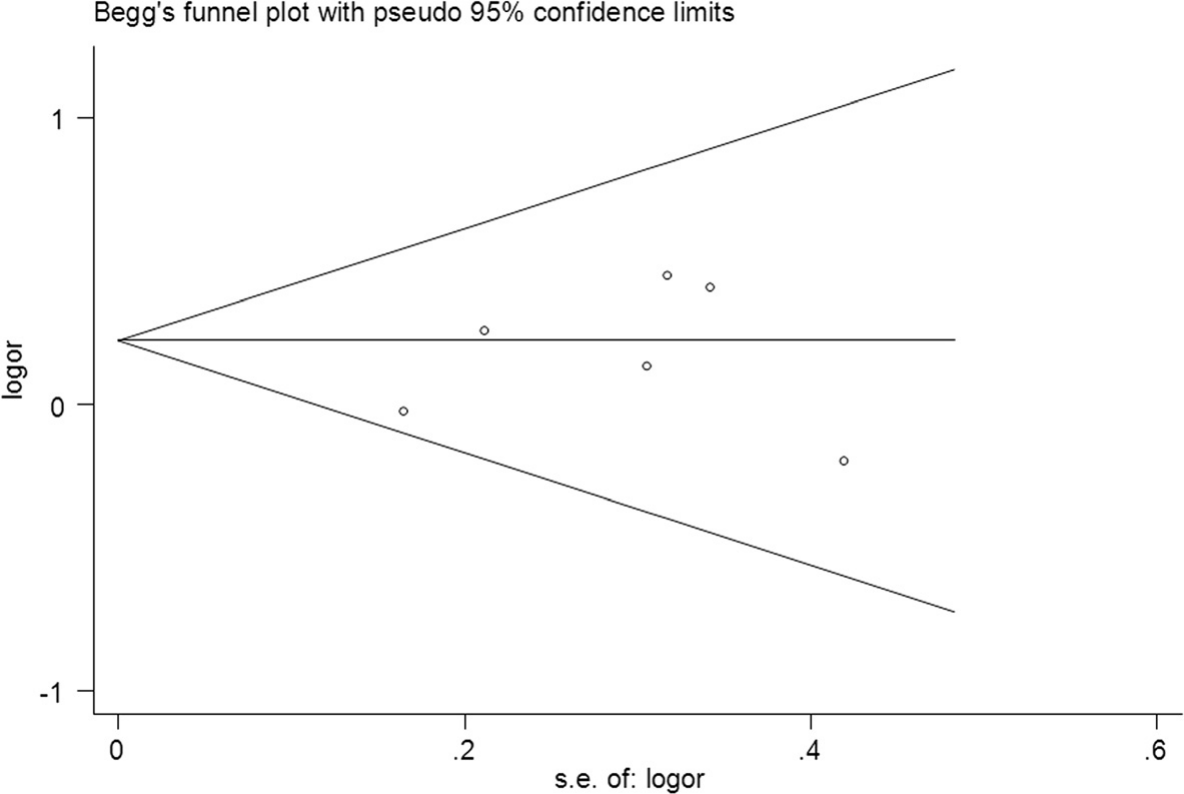
**Funnel plots for publication bias of the meta-analysis on the association between EGF +61A/G polymorphism and gastric cancer risk in the overall populations (dominant model GG + AG vs. AA).**

Begg’s funnel plot and Egger’s test were performed to assess the publication bias of literatures. The shape of the funnel plot did not reveal any evidence of obvious asymmetry (Figure 4). Then, the Egger’s test was used to provide statistical evidence of funnel plot symmetry. All the p values of Egger’s tests were more than 0.05 (GG vs. AA: *P* = 0.298; AG vs. AA: *P* = 0.375; GG + AG vs. AA: *P* = 0.738, Figure 5; GG vs. AG + AA: *P* = 0.826), providing statistical evidence of the funnel plots’ symmetry. The results suggested that publication bias did not present in this study.

## Discussion

The epidermal growth factor (EGF), which was identified as a potent mitogenic peptide, has multiple biological functions including induction of DNA synthesis, proliferation, differentiation, and tumorigenesis of epidermal and epithelial tissues through interaction with its receptor EGFR [30,31]. Mounting evidences have demonstrated that the EGF plays a critical role in malignant transformation, tumor growth and progression [32,33] and over-expression of EGF has been found in advanced gastric cancers [34,35]. Moreover, gastric cancer patients with synchronous expression of EGF and EGFR have been reported to have a poor prognosis [34]. Therefore, EGF has been considered to play a pivotal role in the occurrence and malignant progression of gastric cancer. EGF +61A/G polymorphism is the most common SNP located in the 5′-untranslated region of the EGF gene which has been found influence EGF production or protein expression [11]. It was reported that the +61GG and +61AG genotypes were correlated with significant higher expression of EGF than the +61AA genotype in peripheral blood mononuclear cell lines [11]. It is, therefore, biologically reasonable to hypothesize a potential relationship between the EGF +61A/G polymorphism and gastric cancer. To date, several epidemiological studies have investigated the association between EGF +61A/G polymorphism and gastric cancer risk, but the results remain inconclusive. To derive a precise estimation of the relationship, we performed this meta-analysis. Our meta-analysis based on six case–control studies suggested that the EGF +61A/G polymorphism contributes to increased gastric cancer susceptibility, which was consistent with the hypothesis above.

In the present study, we observed that the EGF +61A/G polymorphism presented a risk factor for gastric cancer in Asian populations, but not in Caucasian populations. The inconsistent results among diverse ethnicities demonstrated different roles of the EGF +61A/G polymorphism on gastric cancer risk in different ethnic genetic backgrounds. Nevertheless, because of the limited number of studies among Caucasians included in this study, the observed association between the EGF +61A/G polymorphism and gastric cancer in Caucasians may be caused by chance, because study with small sample size may have insufficient statistical power to determine a slight effect or may have produced an unstable estimation. In this study, there was only one study for Caucasians concerning the EGF +61A/G polymorphism on gastric cancer risk [19]. Moreover, the genotype distribution in the control population of this study was deviate from HWE. Therefore, the negative results of the Caucasian population should be interpreted cautiously.

In subgroup analysis according to the source of control, statistical significant increased gastric cancer risk was observed in population-based studies, but not in hospital-based studies. The reason may be that the hospital-based studies have inherent selection bias because of the fact that the controls in hospital-based studies may just represent a sample of ill-defined reference population, and may not be representative of the study population or the general population [36]. When stratified according to the study quality, statistical significant increased gastric cancer risk was found in high quality studies, but not in low quality studies. The possible explanation for this discrepancy may be that the existence of recall bias and selection bias in the low quality studies. In addition, genotyping methods without quality control in the studies of low quality should be also considered when deciphering these inconsistent results.

It was possible that the selection bias could have played a role in the present meta-analysis, because the genotype distribution of the EGF +61A/G polymorphism in the control populations deviates from the law of HWE in one of the eligible studies [19]. Previous studies have demonstrated that deviation from the law of HWE may be owing to genetic reasons such as non-random mating, or the alleles reflect recent mutations that have not reached equilibrium, as well as methodological reasons including genotyping errors or biased selection of subjects from the general population [37,38]. Because of the reasons of disequilibrium, the results from genetic association studies might be false if the genotype distribution in the control group was inconsistent with HWE [39]. Therefore, we performed subgroup analysis according to HWE in controls. The results revealed that the increased gastric cancer risk was still evident in studies consistent with HWE, suggesting that the HWE in controls probably had little effect on the overall estimates.

One of the main concerns in a sound meta-analysis is the heterogeneity which exists between studies because heterogeneous data are liable to result in misleading results, and finding the sources of heterogeneity is one of the most important goals in a meta-analysis [40,41]. In the present study, significant between-study heterogeneity was observed in the pooled analyses of total eligible studies (GG vs. AA: *P*_*Q*_ *=* 0.074; GG vs. AG + AA: *P*_*Q*_ *=* 0.020). To identify the sources of heterogeneity, we performed subgroup analyses and meta-regression analysis. Subgroup analyses by source of controls and study quality revealed that the heterogeneity still existed in hospital-based studies and low quality studies. Then we performed meta-regression analysis to further identify the source of heterogeneity. Meta-regression analysis revealed that the HWE in controls was the major source of the heterogeneity. When excluding the HWE-violating study, all P_Q_ values in the overall populations and subgroup analyses were greater than 0.10. Interestingly, the summary ORs in the overall population and subgroup analyses were not materially changed by excluding this study, suggesting that our results were robust and reliable.

Some limitations of this meta-analysis should be mentioned. First, the controls of the eligible studies were not uniformly defined. Although the controls were mainly selected from healthy subjects, some had benign disease such as chronic gastritis, *H. pylori* positive and so on. Therefore, non-differential misclassification bias was possible because these studies may have included the control populations who have different risk of developing gastric cancer. Second, our results were based on unadjusted estimates and a more precise analysis could be conducted if more individual data were available, this would allow for adjustment by other covariates such as the quantity of salty food consumption, drinking, smoking and *H. pylori* infection. Third, the number of studies included in the meta-analysis for Caucasian population was relatively small and there was only one study in the Caucasian group, which may lead to insufficient statistical power and generated a fluctuate estimation.

## Conclusions

Despite the limitations, this meta-analysis strongly suggests that the EGF +61A/G polymorphism contributes to increased gastric cancer susceptibility, especially in Asian populations. Further studies with large sample size and well design in diverse ethnicities should be conducted to further investigate the association.
